# Associations between Schizotypal Facets and Symptoms of Disordered Eating in Women

**DOI:** 10.3390/ijerph191811157

**Published:** 2022-09-06

**Authors:** Viren Swami, David Barron, Adrian Furnham

**Affiliations:** 1 School of Psychology and Sport Science, Anglia Ruskin University, Cambridge CB1 1PT, UK; 2 Centre for Psychological Medicine, Perdana University, Kuala Lumpur 50490, Malaysia; 3 Department of Leadership and Organizational Behaviour, Norwegian Business School, 0484 Oslo, Norway

**Keywords:** schizotypy, disordered eating, excessive social anxiety, drive for thinness, body dissatisfaction, bulimia symptoms

## Abstract

Research has suggested that schizotypy—a personality organisation representing latent vulnerability for schizophrenia-spectrum disorders—may be elevated in women with symptoms of disordered eating. However, studies have not fully considered associations between symptoms of disordered eating and multidimensional schizotypy. To overcome this limitation, we asked an online sample of 235 women from the United States to complete measures of symptoms of disordered eating (drive for thinness, body dissatisfaction, and bulimic symptoms) and multidimensional schizotypy. Correlational analyses indicated significant associations between drive for thinness and bulimic symptoms, respectively, and most schizotypal facets. Body dissatisfaction was significantly associated with only two schizotypal facets. Overall, the strength of correlations was weak-to-moderate. Regression results indicated that only the schizotypal feature of excessive social anxiety was significantly associated with all risk for disordered eating factors. These results are consistent with aetiological models of disordered eating that highlight socio-affective difficulties as risk factors for symptoms of disordered eating.

## 1. Introduction

According to the *Diagnostic and Statistical Manual of Mental Disorders* (*DSM-5*) [1], eating disorders are characterised by a severe and chronic disturbance in eating behaviour, which causes significant impairment [2,3]. They are a major area of concern for clinicians, families, and wider society given their detrimental impacts on health and psychological well-being, as well as their wide-ranging socioeconomic costs [4]. Additionally, eating disorders have some of the highest rates of mortality of all psychiatric disorders [5]. It is, therefore, imperative that researchers and practitioners seek to understand risk factors for the development of symptoms of disordered eating and thereby contribute to the development of effective intervention strategies [6,7]. The current consensus on the causes of eating disorders suggests that the aetiology is complex and that there are likely multiple causal factors that give rise to the various symptoms of disordered eating [8].

One strand of research has focused on the role of personality features in the aetiology of symptoms of disordered eating [9,10]. In particular, personality disorders are frequently comorbid with eating disorders [11], with some studies suggesting that comorbidity may reach 60% [12]. In explanation, it has been suggested that the dysregulation (e.g., affective lability, interpersonal dysfunction, and behavioural disinhibition) that accompanies personality disorders has an influence on the aetiology and maintenance of disordered eating [13]. While much of this work has focused on comorbidities with obsessive-compulsive personality disorder [14] and borderline personality disorder [15], more recent studies have argued for a focus on broader comorbid personality psychopathology [16].

Within this broader focus, some relevant research has suggested that schizophrenia-spectrum disorders may also be comorbid with eating disorders [17,18]. For instance, in very early work, both Kraepelin [19] and Bleuler [20] described disorganised and uncontrolled food intake as a characteristic symptom of schizophrenia. This line of thinking has been picked up more recently, with Sansone and Sansone [21] suggesting that childhood trauma may heighten the risk for both schizophrenia-spectrum disorders and eating disorders, indicative of complex inter-relationships between these constructs. Meanwhile, the evidence base linking schizophrenia-spectrum disorders and symptoms of disordered eating is more equivocal, with some studies identifying a significant association [22] and others suggesting that the association does not reach significance [23]. Even so, a recent review concluded that rates of binge eating and disordered eating behaviours were elevated in people with schizophrenia-spectrum disorders, although many studies relied on clinical samples and did not use valid assessment tools [24].

One issue in interpreting the links between schizophrenia-spectrum disorders and disordered eating symptoms is that, in clinical populations at least, anti-psychotic medication may lead to changes in dietary habits [25]. One way of getting around this issue is to focus on the construct of *schizotypy*, a heterogeneous personality organisation representing latent vulnerability for schizophrenia-spectrum disorders [26]. Focusing on schizotypy is consistent with continuum models of mental illness [27], wherein it is suggested that the phenotypic manifestation of schizotypal traits can occur in the general population without necessarily being associated with schizophrenia-spectrum personality disorders or full psychosis [28,29,30]. Moreover, longitudinal studies have suggested that the manifestation of schizotypal traits is a risk factor for transitioning from subclinical symptomatology to full-blown psychosis [31], particularly in the presence of genetic or environmental risk factors [28,32]. In other words, schizotypy can be viewed as an important bridge to the onset of psychosis in general and schizophrenia in particular [26].

Importantly, schizotypy and schizophrenia-spectrum disorders are not isomorphic [26], which makes it important to examine associations between schizotypy and symptoms of disordered eating. Indeed, in developing the construct of schizotypy, Rado [33] described a kinaesthetic predisposition that results in an aberrant awareness of the body, which in turn led to schizotypic body image distortions. Similarly, Meehl [34] described a range of schizotypal signs and symptoms, including body image aberrations, a suggestion that has received support more recently in the finding that high schizotypal traits are associated with sense of detachment from one’s lived body and that abnormal bodily experiences may be a marker of schizophrenia proneness [35]. Other studies have also suggested that body ownership (i.e., a component of the bodily self) as measured using the Rubber Hand Illusion is significantly associated with schizotypy, indicating that body image disturbances may be central to prodromal schizophrenia [36,37].

An alternative way to approach this topic is to assess associations between symptoms of disordered eating and schizotypy, which two previous studies have done. First, Raynal and colleagues [16] reported that, in French college students (*N* = 378), those who were assessed as having disordered eating behaviours had significantly higher schizotypy scores than those without disordered eating behaviours. One important limitation of this study, however, is the fact that the authors only examined global schizotypy scores, which contrasts with the consensus among schizotypy scholars that the construct is heterogeneous and multidimensional [30,38]. More specifically, there is a wealth of evidence to suggest that schizotypy is characterised by a positive or psychotic-like dimension (characterised by disruptions in content of thought, perceptual oddities, and suspiciousness or paranoia), a negative or deficit dimension (characterised by diminished experiences, such as anergia and anhedonia), and a disorganised dimension (characterised by disturbances in the ability to organise and express thoughts and behaviours) [26].

An earlier study did examine associations between multidimensional schizotypy and disordered eating attitudes [39]. Here, it was reported across two studies that several facets of schizotypy—particularly ideas of reference, odd beliefs/magical thinking, and suspiciousness—were significantly and positively associated with disordered eating attitudes in university students from the United States, although effects sizes were weak-to-moderate. This suggests that the positive or psychotic-like facets of schizotypy may be associated with greater disordered eating attitudes, which would be consistent with the finding that positive schizotypy is associated with a weakened sense of body ownership [37]. However, issues in interpreting these findings relate to the small sample sizes in the studies by Bremser and Gallup [39] (*N* = 42 and 37, respectively), the sole focus on correlational analyses (i.e., no follow-up analyses were conducted), and the lack of consideration of possible gendered effects (i.e., data were pooled across women and men for analyses).

In view of these issues, the present study sought to make an incremental contribution to knowledge by re-examining associations between multidimensional schizotypal facets and symptoms of disordered eating in a larger sample of women. While this study adopted a largely exploratory framework given the dearth of relevant research, several schizotypal facets stand out as *prima facie* antecedents of disordered eating. These include the positive schizotypal facets identified by Bremser and Gallup [39], namely ideas of reference, odd beliefs/magical thinking, and suspiciousness. Two other potential candidates were unusual perceptual experiences (which may be associated with unrealistic perceptions of one’s body) and constricted affect (which may be associated with the behavioural inhibition that is common in disordered eating [40]. Finally, we also hypothesised a role for the schizotypal trait of excessive social anxiety, which refers to excessive self-consciousness, social fear, and fear of social interaction and judgement. These socio-affective difficulties have been highlighted as risk factors for disordered eating [41], with previous studies indicating that non-schizotypal social anxiety uniquely predicts symptoms of disordered eating in women [42].

## 2. Materials and Methods

### 2.1. Participants

Participants were 235 women from the United States, who ranged in age from 19 to 70 years (*M* = 39.09, *SD* = 13.07) and in body mass index (BMI) from 15.24 to 49.92 kg/m^2^ (*M* = 24.92, *SD* = 6.44). The majority of people in the sample were White (75.3%), with 10.2% of African American descent, and the remainder of other racial groups.

### 2.2. Measures

#### 2.2.1. Schizotypy

Participants completed the 74-item Schizotypal Personality Questionnaire (SPQ) [43], designed to measure nine schizotypal factors, namely No Close Friends, Constricted Affect, Ideas of Reference, Odd Beliefs and Magical Thinking, Unusual Perceptual Experiences, Odd or Eccentric Behaviour, Odd Speech, Suspiciousness, and Excessive Social Anxiety. Items were responded to using a dichotomous scale (*yes*/*no*). Each *yes* response counts as one point and the nine factor scores were computed as the mean of all items associated with each subscale. Scores on the SPQ have very good factorial validity, adequate reliability, and good patterns of convergent and divergent validity (Raine, 1991). In the present study, McDonald’s omega was ≥ 0.72 for all subscales.

#### 2.2.2. Disordered Eating

Participants completed three subscales of the Eating Disorders Inventory-3 (EDI-3) [44], namely Drive for Thinness (EDI-DT), Bulimic Symptoms (EDI-BS), and Body Dissatisfaction (EDI-BD). Scores on these subscales perform well as prognostic indicators of risk for disordered eating in women [45]. All items were rated on a 6-point scale (1 = *never*, 6 = *always*) and subscale scores were computed as the mean of relevant items. The reliability and validity of EDI-3 scores are well-established [45]. Here, McDonald’s omega was ≥ 0.77 for all subscales.

#### 2.2.3. Demographics

Participants provided their demographic details consisting of age, race, height, and weight. Self-reported height and weight data were used to compute BMI as kg/m^2^.

#### 2.2.4. Procedures

The project was approved by the relevant departmental ethics committee. Data were collected via Amazon’s Mechanical Turk (MTurk) website, a crowdsourcing Internet marketplace that allows individuals to complete academic surveys for monetary compensation. To be eligible, MTurk workers had to be United States citizens of adult age, fluent in English, have achieved at least a 98% approval rate, and completed at least 1000 Human Intelligence Tasks (HITs). The project was advertised as a study on “eating habits and personality” and included an estimated duration. After providing digital informed consent, participants were directed to the measures described above, which were presented in an anonymous form and in random order via the randomisation function with Qualtrics, which hosted the survey. The survey included an attention check item, which needed to be answered correctly for participants to be included in the analyses. To further increase confidence in the validity of our data, we also screened for random responding and for batches of responses obtained at the same exact time and excluded responses from surveys completed in under 10 min. In exchange for completing the survey, participants were paid USD 1.50. All participants received written debriefing information at the end of the survey.

#### 2.2.5. Statistical Analyses

All analyses were conducted in SPSS Statistics version 28. There were no missing data in the final dataset and initial analyses indicated that all assumptions for multiple regression analyses were met. We first computed bivariate correlations between the EDI-3 subscale scores and the schizotypal facets. Due to the large number of comparisons, a Bonferroni correction was applied such that *p* = 0.05/11 = 0.005. The sample size (*N* = 235) was sufficient to detect whether a correlational coefficient differs from zero when α = 0.05, β = 0.20, and expected *r* = 0.20 [46]. Next, we conducted three multiple linear regression analyses, with each of the EDI-3 factor scores entered as criterion variables, and all schizotypal variables entered as predictors. Multicollinearity was not a limiting factor (variance inflation factors ≤ 3.04). We also conducted hierarchical multiple regressions with age, race, and BMI included in a first step and the SPQ facets included in a second step, but this did not alter the pattern of regression results *vis-à-vis* schizotypal facets. For parsimony, we report the results of the multiple linear regression here.

## 3. Results

Descriptive statistics and bivariate correlations between all variables are reported in Table 1. As can be seen, drive for thinness was significantly and positively associated with scores on all but one (odd beliefs and magical thinking) of the SPQ facets, with associations generally being weak-to-moderate in strength. Body dissatisfaction was only significantly and positively associated with excessive social anxiety and unusual perceptual experiences, although the strength of the relationships was weak. Finally, bulimic symptoms scores were significantly associated with scores on all but one SPQ facet (odd beliefs and magical thinking), although the associations were weak.

The first regression with drive for thinness was significant, *F*(9, 234) = 5.16, *p* < 0.001, Adj. *R*^2^ = 0.14, but the only significant predictor was excessive social anxiety (B = 0.17, SE = 0.04, β = 0.35, *t* = 4.27, *p* < 0.001). The second regression with bulimic symptoms was also significant, *F*(9, 234) = 5.02, *p* < 0.001, Adj. *R*^2^ = 0.13, with excessive social anxiety again emerging as the only significant predictor (B = 0.10, SE = 0.03, β = 0.28, *t* = 3.41, *p* = 0.001). The final regression with body dissatisfaction was likewise significant, *F*(9, 234) = 2.28, *p* = 0.018, Adj. *R*^2^ = 005, with excessive social anxiety being the only significant predictor (B = 0.06, SE = 0.03, β = 0.17, *t* = 2.04, *p* = 0.042).

## 4. Discussion

The results of this study showed that only the schizotypal facet of excessive social anxiety emerged as a significant predictor of symptoms of disordered eating in a sample of women from the United States. Additional schizotypal facets were significantly correlated with symptoms of disordered eating—particularly drive for thinness and bulimic symptoms—which is broadly consistent with the earlier findings of Bremser and Gallup [39]. Importantly, however, these facets did not emerge as significant predictors once the variance accounted for by excessive social anxiety was considered. Put differently, of the nine schizotypal facets, excessive social anxiety appears to be the dominant correlate of symptoms of disordered eating, a finding that may have been obscured in previous work. More generally, the present results corroborate previous work showing that women with disordered eating symptoms have higher schizotypal scores compared to women in a control group [16].

Our results are consistent with aetiological models of disordered eating, which highlight social anxiety as a key risk factor for the development of eating pathology in adults [41]. While the role of social anxiety in the aetiology of disordered eating is well-documented, our results are important in their own right in highlighting that excessive social anxiety—a core facet of schizotypy—places women at heightened risk for symptoms of disordered eating. This may occur through a number of possible routes. For example, it is possible that higher excessive social anxiety leads women to develop unhealthy anxiety over their appearance, experience shame in social situations, or develop pathological fear of negative appearance-based evaluations [42]. In this view, excessive social anxiety can be conceptualised as a risk factor for the development of disordered eating symptoms, such that anxiety around how one appears to others may lead to excessive preoccupation with appearance and body image that, in turn, heightens the risk for developing symptoms of disordered eating. 

Another possibility is that excessive social anxiety may be secondary to symptoms of disordered eating (i.e., a consequence of disordered eating psychopathology). For instance, it may be that individuals who are high in symptoms of disordered eating are more likely to interpret social situations or relationships as threatening or unmanageable, leading to excessive social anxiety [47]. While our data are cross-sectional and cannot distinguish between these theoretical accounts, other work examining the temporal relations between social anxiety and disordered eating symptoms have generally lent support to the notion that social anxiety precedes the development of symptoms of disordered eating [48,49].

A not unrelated possibility is that excessive social anxiety—and schizotypal facets more generally—may share common vulnerability factors with symptoms of disordered eating. For instance, it may be that there are factors that increase vulnerability to both excessive social anxiety and symptoms of disordered eating. One such factor is perfectionism, which has been found to significantly elevated in both individuals with eating disorders and social anxiety disorder [50]. Indeed, perfectionism has been found to be significantly associated with both social anxiety and disordered eating symptoms in non-clinical women [51]. The cross-sectional nature of the present study means that we cannot rule out any of these possibilities, although it seems possible that all routes will shape the incidence of disordered eating in women to a lesser or greater degree. Furthermore, it should be noted that the variance explained by all schizotypal facets in the regressions was generally low (Adj. *R*^2^ = 0.05–0.14), suggestive of weak predictive associations.

One limitation of the present work was the reliance on an online sample of women across a wide age range, which may mean that our findings lack generalisability. Indeed, our findings should be considered limited to women from the United States, and replications of our findings in other cultural contexts may be useful. In a similar vein, it would be useful to examine the extent to which the present findings are robust when samples of men are included. In addition, the present study operationalised schizotypy using the SPQ and, although this is a widely used measure, there may be some utility in using more recent measures of multidimensional schizotypy in future research [52]. Additionally, because we did not assess other comorbidities that are known to co-occur with eating disorders (e.g., obsessive-compulsive disorder) or early onset psychosis, these may have acted as natural confounds in our analyses. In a similar vein, given the focus of the present study on direct associations between schizotypal facets and symptoms of disordered eating, we did not include additional variables that may mediate relationships reported here. In future work, there may be value in including such mediating variables, as this would help scholars better understand possible mechanistic pathways that lead from excessive social anxiety and schizotypy more generally to symptoms of disordered eating.

## 5. Conclusions

Despite these limitations, our results help clarify earlier reports that schizotypy is elevated in women with disordered eating symptomatology [16,39]. Specifically, our results suggest that it is the schizotypal feature of excessive social anxiety that may most prominently place women at heightened risk for symptoms of disordered eating. Future studies could extend our work through the use of longitudinal designs that test the causational effects of multidimensional schizotypy on symptoms of disordered eating. Additionally, future research would do well to better understand the role that schizotypal facets other than excessive social anxiety play in the aetiology of symptoms of disordered eating. To the extent that these facets play a role in the course and treatment outcomes of disordered eating symptoms, further research in this area would be vital for generating new insights into illness pathways and possible transdiagnostic factors.

## Figures and Tables

**Table 1:** Descriptive statistics and bivariate correlations between all variables included in the present study.

	(1)	(2)	(3)	(4)	(5)	(6)	(7)	(8)	(9)	(10)	(11)	(12)
(1) EDI-DT		0.30 *	0.62 *	0.24 *	0.37 *	0.07	0.21 *	0.26 *	0.19 *	0.20 *	0.20 *	0.24 *
(2) EDI-BD			0.25 *	0.20 *	0.19 *	0.10	0.18	0.08	0.05	0.08	0.12	0.09
(3) EDI-BS				0.28 *	0.33 *	0.17	0.29 *	0.28 *	0.21 *	0.25 *	0.24 *	0.24 *
(4) SPQ-IoR					0.30 *	0.47 *	0.61 *	0.44 *	0.29 *	0.44 *	0.32 *	0.69 *
(5) SPQ-ESA						0.04	0.31 *	0.40 *	0.63 *	0.43 *	0.53 *	0.44 *
(6) SPQ-OBoMT							0.59 *	0.39 *	0.18	0.28 *	0.17	0.42 *
(7) SPQ-UPE								0.58 *	0.42 *	0.58 *	0.49 *	0.58 *
(8) SPQ-OoEB									0.43 *	0.61 *	0.50 *	0.55 *
(9) SPQ-NCF										0.47 *	0.72 *	0.59 *
(10) SPQ-OS											0.60 *	0.55 *
(11) SPQ-CA												0.58 *
(12) SPQ-Sus												
*M*	3.22	3.00	2.10	2.52	4.63	1.26	1.77	2.00	3.89	2.67	2.30	2.43
*SD*	1.37	0.57	1.02	2.56	2.92	1.73	2.00	2.28	2.71	2.47	2.22	2.38

*Note*. *N* = 235. * significant at Bonferroni-corrected *p* = 0.005. EDI = Eating Disorders Inventory-3; DT = Drive for Thinness; BD = Body Dissatisfaction; BS = Bulimic Symptoms; IoR = Ideas of Reference; ESA = Excessive Social Anxiety; OBoMT = Odd Beliefs or Magical Thinking; UPE = Unusual Perceptual Experiences; OoEB = Odd or Eccentric Behaviour; NCF = No Close Friends; OS = Odd Speech; CA = Constricted Affect; Sus = Suspiciousness.

## Data Availability

Data is available from the corresponding author.
